# Triage Accuracy and the 2015 Field Trauma Triage Criteria Update

**DOI:** 10.1001/jamanetworkopen.2025.52092

**Published:** 2026-01-05

**Authors:** Bourke W. Tillmann, Avery B. Nathens, Matthew P. Guttman, Corey Freedman, Priscila Pequeno, Damon C. Scales, Petros Pechlivanoglou, Barbara Haas

**Affiliations:** 1 Interdepartmental Division of Critical Care, University of Toronto, Toronto, Ontario, Canada; 2 Department of Medicine, Division of Respirology and Critical Care Medicine, University Health Network, Toronto, Ontario, Canada; 3 Tory Trauma Program, Sunnybrook Health Sciences Centre, Toronto, Ontario, Canada; 4 Department of Surgery, University of Toronto, Toronto, Ontario, Canada; 5 ICES, University of Toronto, Toronto, Ontario, Canada; 6 Toronto Health Economic and Technology Assessment Collaborative, Toronto, Ontario, Canada; 7 The Hospital for Sick Children, Toronto, Ontario, Canada

## Abstract

**Importance:**

Despite evidence demonstrating that severely injured patients treated at trauma centers have improved outcomes relative to those treated at nontrauma centers, rates of undertriage (transportation of severely injured patients to nontrauma centers) remain high. While national expert panels have developed field trauma triage (FTT) guidelines to aid in the triage of injured patients, the impact of the implementation of these guidelines on existing triage patterns is unknown.

**Objective:**

To examine the association between the 2015 update to FTT guidelines and trends in trauma triage in a mature trauma system in Ontario, Canada.

**Design, Setting, and Participants:**

This retrospective, population-based cohort study was conducted from April 1, 2009, to March 31, 2020, among individuals aged at least 16 years who presented to hospital with a traumatic injury in Ontario, Canada. Statistical analysis was conducted from October 2023 to October 2025.

**Exposure:**

Implementation of the 2015 update to the FTT guidelines as specified in version 2.1 of the Ontario Basic Life Support Patient Care Standards. The exposure was divided into 3 timeframes: pre-FTT (April 1, 2009, to June 30, 2014), implementation (July 1, 2014, to May 2 31, 2015), and postimplementation (June 1, 2015, to March 31, 2020).

**Main Outcomes and Measures:**

Patients were categorized as undertriaged, overtriaged, or appropriately triaged based on hospital of presentation and presence of a severe injury (defined as Injury Severity Score [ISS] ≥16 or death within 24 hours of injury). Interrupted time series analysis was used to estimate temporal trends in triage rates and variations in these rates associated with the updated FTT guidelines, adjusting for differences in sociodemographic and injury characteristics.

**Results:**

Of 281 268 patients with traumatic injury (mean [SD] age, 62.4 [22.5] years; 141 450 [50.3%] female; median [IQR] ISS, 4 [4-9]) included, 53 870 (19.2%) presented directly to a trauma center. Among patients who presented to a trauma center, 28 494 (52.9%) had a minor injury. Population-level rates of undertriage and overtriage were 63.5% and 12.3%, respectively. At the population level, the implementation of updated FTT guidelines was associated with a 15.2% instantaneous decrease in undertriage (rate ratio [RR], 0.85; 95% CI, 0.77-0.94). Conversely, there was no instantaneous change in overtriage (RR, 0.90; 95% CI, 0.79-1.04). During the 5 years after implementation, rates of undertriage increased by 2.4% per year (RR, 1.02; 95% CI, 1.02-1.03) while overtriage decreased by 3.8% annually (RR, 0.96; 95% CI, 0.95-0.97). Across trauma centers, the updated FTT guidelines were associated with a 14.4% instantaneous decrease in the proportion of patients with minor injuries presenting to a trauma center (RR, 0.86; 95% CI, 0.77-0.95). During 5 years after implementation, the rate at which patients with minor injuries presented directly to a trauma center remained constant (RR, 1.00; 95% CI, 0.99-1.01).

**Conclusion and Relevance:**

In this cohort study, the implementation of updated FTT guidelines was immediately associated with a 15% decrease in undertriage without an increase in overtriage. After the implementation of these guidelines, rates of undertriage increased by 2% each year. These findings suggest that FTT is effective in decreasing undertriage; however, ongoing monitoring is required to ensure that these benefits are maintained.

## Key Points

**Question:**

Was the implementation of updated field trauma triage (FTT) guidelines on triage practices associated with changes in rates of overtriage or undertriage in a large, regional trauma system?

**Findings:**

In this population-based cohort study of 281 268 injured patients, the implementation of updated FTT guidelines was associated with a 15.2% instantaneous decrease in the rate of undertriage. However, during the 5 years after the implementation of the new guidelines, rates of undertriage increased by 2.4% annually.

**Meaning:**

These findings suggest that while implementation of updated FTT guidelines was associated with a decrease in undertriage, ongoing monitoring is required to ensure that the benefits associated with new guidelines are maintained.

## Teaser

This cohort study examines the association between the 2015 update to field trauma triage guidelines and trends in trauma triage in Ontario, Canada.

## Introduction

Severely injured patients transported directly to a trauma center are 30% less likely to die within the first 48 hours than those transported to nontrauma centers.^1^ Nonetheless, undertriage (the transportation of severely injured patients to nontrauma centers) remains common.^2,3^ Prehospital triage is challenging; there is minimal time to determine injury severity, diagnostic information is limited, and most patients have minor injuries that do not require trauma center care.^4^ National expert panels have developed field trauma triage (FTT) guidelines to help prehospital personnel identify patients likely to have severe injuries.^5,6^ While FTT criteria can accurately identify severely injured patients, how implementation of updated FTT guidelines alters triage patterns in a well-established trauma system is unknown.^7,8^

Multiple jurisdictions with long-standing FTT guidelines continue to report high rates of undertriage.^2,3,9,10,11^ Hypotheses for these high rates include a lack of geographic access to trauma centers, inadequate familiarity with triage guidelines, implicit biases related to age and gender, and limited experience in treating injured patients.^12,13,14,15,16,17^ Given that a lack of experience and implicit bias have been identified as potential drivers of undertriage, it is unclear to what extent the existence or modification of FTT guidelines can impact triage practices. In other words, it is unclear whether these guidelines increase the identification of severely injured patients or only direct emergency medical service (EMS) personnel to transport patients they already perceive as severely injured to trauma centers. To this end, the goal of this study was to analyze the association between updates to FTT guidelines and triage practices in a large, regional trauma system.

## Methods

### Study Design

This was a retrospective, population-based cohort study that used interrupted time series analyses to examine the association between updated FTT guidelines and triage patterns of injured adults. This study was approved by the ICES Privacy and Compliance Office with a waiver of informed consent because ICES is a prescribed entity under Personal Health Information Protection Act and the Coroners Act. This status allow ICES to conduct analyses and compile statistical information about the management and effectiveness of the health system and the health or safety of the public. This study is reported following the Reporting of Studies Conducted Using Observational Routinely Collected Data (RECORD) statement, an extension of the Strengthening the Reporting of Observational Studies in Epidemiology (STROBE) reporting guideline developed for observational studies conducted using routinely-collected health data.^18,19^

### Setting

This study was conducted in Ontario, Canada, between April 1, 2009, and March 31, 2020. Ontario is Canada’s most populous province (population, 14.6 million) and encompasses an area of 415 000 square miles (approximately 1.5 times the size of Texas). The first trauma center in Ontario was established in 1976.^20^ Currently, its regional health care system has 217 acute care hospital sites, including 9 provincially designated adult lead trauma hospitals.^12,21^ More than 80% of the population lives within 1 hour of a lead trauma hospital.^22^ Prehospital care is provided by 55 government-designated delivery agents for land ambulance and 1 air ambulance service.^23,24^ All paramedics must complete an approved training program and receive medical delegation and oversight through 1 of 7 regional base hospitals. There is 1 provincial set of paramedic practice standards, which includes provisions for FTT.^25^

### Data Sources

Data were derived from administrative datasets held at ICES. Patients were identified using the National Ambulatory Care Reporting System and Discharge Abstract Database. Details of the datasets are available in eAppendix 1 in Supplement 1 and have been previously described.^1,26,27^ ICES datasets were linked using unique encoded identifiers and analyzed at ICES.

### Study Participants

We identified all individuals aged at least 16 years who presented to an emergency department (ED) secondary to a traumatic injury. Traumatic injuries were identified by the presence of *International Statistical Classification of Diseases and Related Health Problems, Tenth Revision *(*ICD-10*) diagnosis codes in the range S1.0-T14.9. We excluded Ontario nonresidents, patients missing age, sex, or home address data, and those who left the ED without being seen or against medical advice. Additionally, we excluded patients with environmental injuries and older adults (age ≥65 years) with isolated hip fractures, as these patients are not triaged using the same guidelines as patients with other traumatic injuries. To eliminate patients who presented for sequelae of a previous injury, we excluded patients who had any injury-related ED visit in the 90 days prior to the index event or an admission for a severe injury in the prior year. To ensure that our study was not confounded by patients with minor injuries (to whom FTT criteria do not apply) we also excluded patients who either were discharged directly home from the ED of the nontrauma center to which they presented, regardless of the calculated Injury Severity Score (ISS), or, despite being transferred between nontrauma centers, did not have a severe injury and were not admitted.

### Patient Characteristics

Patients were characterized by age, sex, comorbidity, frailty, receipt of long-term home care, nursing home residence, location of residence, and socioeconomic status. Adjusted Clinical Groups were used to identify comorbidity level and frailty.^28,29^ Location of residence was categorized as urban or rural based on the rural and small town definition used by Statistics Canada.^30^ Socioeconomic status was defined using the Ontario Marginalization Index.^31,32^ Injuries were characterized based on ISS, presence of a severe head, chest, or abdominal injury, mechanism of injury (MOI), date and time of presentation, and triage acuity.^33,34,35^ ISS were calculated using an validated algorithm to derive Abbreviated Injury Scale scores from *ICD-10* diagnosis codes.^36^ As early deaths limit full injury ascertainment, if a patient died within 24 hours of presentation and their ISS was below 15, they were assigned to the highest ISS category. Details of all characteristics are available in eAppendix 1 in Supplement 1.

We dichotomized patients as having a severe or minor injury. If a patient had an ISS of at least 16 or died within 24 hours of presentation, they were identified as having a severe injury. All other patients were categorized as having a minor injury. We also identified patients with a high-risk injury independent of ISS. These patients included those who had a critical injury (as defined by the American College of Surgeons, as shown eAppendix 1 in Supplement 1), received mechanical ventilation in an ED, or received blood within 24 hours of presentation.^37,38^

### Exposure

The exposure of interest was implementation of the 2015 update to the FTT guidelines as specified in version 2.1 of the Ontario Basic Life Support Patient Care Standards.^39^ This version of the FTT guidelines was based on the 2011 Recommendations of the National Expert Panel on Field Triage.^5^ However, the 2015 Ontario guidelines did not include ground-level falls in older adults as a special consideration for trauma center transport. Prior to the rollout of updated FTT guidelines, triage decisions were based on those included in version 2 of the provincial Basic Life Support Patient Care Standards.^40^ The new guidelines made significant changes to the physiologic, anatomic, and mechanistic criteria used to identify patients who required transport to a trauma center and added special circumstances requiring lower thresholds for transport (Table 1).

**Table 1.** Comparison of Trauma Triage Criteria Indicating That an Injured Adult Should Be Transported Directly to a Trauma Center Prior to and After the Implementation of the 2015 Update to the Field Trauma Triage Criteria

Criteria	Preimplementation guidelines	Postimplementation guidelines
Transport time	If <30 min, bypass local hospitals and transport to nearest trauma center If >30 min, transport by air ambulance to trauma center when available	If <30 min, bypass local hospitals and transport to nearest trauma center (may be extended to 60 min in specific scenarios) If >30 min, transport by air ambulance to trauma center when available and air travel time is less than land time
Physiologic criteria	GCS ≤10 If GCS >10 any 2 of: Altered level of consciousness HR <50 or >120 bpm SBP <80 mm Hg or absent radial pulse RR <10 or >24 breaths per min	GCS <14 SBP <90 mm Hg RR<10 or ≥30 breaths per min or need for ventilatory support
Anatomic	Penetrating injury to head, neck, trunk, or groin Amputation above wrist or ankle Spinal cord injury with paraplegia or quadriplegia	Penetrating injury to head, neck, trunk, or proximal to elbows or knees Chest wall instability or deformity (eg, flail chest) ≥2 Proximal long-bone fractures Crusted, degloved, mangled, or pulseless extremity Amputation proximal to wrist or ankle Pelvic fractures Open or depressed skull fracture Paralysis
Mechanism	Fall >5 m (16.4 ft) MVC >30 km/h (18.6 mi/h) or death of a co-occupant Person ejected from vehicle at speed >30km/h (18.6 mi/h) MVC rollover with unbelted occupant Vehicle struck fixed object (eg, rock cut, tree, pole) or large animal (eg, moose, deer, bear) Pedestrian struck by vehicle >15km/h (9.3 mi/h)	Fall ≥6 m (19.7 ft) MVC with intrusion ≥0.3 m (1 ft) on patient’s side or ≥0.5 m (1.6 ft) at any site Person ejected from vehicle MVC with death in the same passenger compartment MVC with telemetry data consistent with high-risk injury Motorcycle crash ≥30 km/h (18.6 mi/h) Pedestrian or bicyclist thrown, run over, or struck by vehicle ≥30 km/h (18.6 mi/h)
Diversion	Uncomfortable with safety of patient during transport Concern patient will not survive transport to nearest trauma center Despite the above, VSA patients with penetrating thoracoabdominal injuries may bypass local EDs to facilitate transportation to nearest trauma center	Unable to secure airway Patient unlikely to survive transport to trauma center Despite the above, transport patients to nearest trauma center if they meet all the following: Penetrating torso, head, or neck injury VSA but do not meet TOR criteria Transport to the trauma center <30 min
Special criteria suggesting lower threshold for transport to trauma center	None	Age >55 y Age >65 y and SBP <110 mm Hg Using anticoagulants or having a bleeding disorder Combined burns and trauma ≥20 wk pregnant

Abbreviations: bpm, beats per minute; ED, emergency department; GCS, Glasgow Coma Scale; HR, heart rate; MVC, motor vehicle collision; NA, not applicable; RR, respiratory rate; SBP, systolic blood pressure; TOR, termination of resuscitation; VSA, vital signs absent.

The updated FTT guidelines were announced on June 23, 2014, and all ambulance services were mandated to implement them by June 1, 2015. To facilitate implementation of the updated FTT guidelines, in June 2014, the Ministry of Health electronically published the revised FTT Standard. In a memorandum from the Director of the Emergency Health Services to the EMS chiefs, municipal chief administrative officers, and Ornge, information regarding the revised standard and expectations for dissemination and training to their personnel was outlined. To support the update, a training bulletin was drafted with information about the revised standard and how it was to be followed. The bulletin was printed by the Ministry of Health and made available to all paramedic services should they wish to distribute it to the frontline staff, or alternatively, provided in electronic format. Laminated copies of the flow diagram were also made available. Paramedic services further educated their staff using the training bulletin or through additional means, such as in-person annual continuing medical education or online modules.

Given the 1-year training and rollout period, the exposure was divided into 3 timeframes: pre-FTT (April 1, 2009, to June 30, 2014), implementation (July 1, 2014, to May 31, 2015), and postimplementation (June 1, 2015, to March 31, 2020). The decision was made to not extend the study cohort past March 2020, as the first wave of the COVID-19 pandemic started in Ontario on March 20, 2020, substantially altering hospital resource availability and triage patterns.^41^ Consequently, associations between subsequent updates to FTT guidelines, based on the 2021 Recommendations of the National Expert Panel on Field Triage, were not examined in this analysis.^6^

### Outcomes

The primary outcome was presentation to a trauma center as the first treating hospital (ie, primary triage). As level III trauma centers were not formally identified in Ontario until 2021, all patients identified as presenting to a trauma center presented to a provincially designated lead trauma hospital.^42^ Patients were then classified as undertriaged or overtriaged based on injury severity. If a patient had a severe injury and was not transported to a trauma center, they were categorized as undertriaged. Conversely, if a patient had a minor injury and presented to a trauma center, they were categorized as overtriaged.

Understanding that patients with high-risk injuries who did not meet severe injury criteria could initially be managed at either a trauma or nontrauma center, all patients with high-risk injuries who survived more than 24 hours and had an ISS less than 16 were classified as appropriately triaged regardless of center of presentation. This decision was made to bias our study in favor of the EMS practitioner making the triage decision.

### Statistical Analysis

Baseline characteristics were summarized using descriptive statistics and compared between patients injured before and after the implementation of FTT.^43^ To evaluate temporal trends in triage we calculated the unadjusted estimated annual percentage change (EAPC) for the rate of each outcome of interest using modified Poisson regression.^44^ Modified Poisson regression was also used to calculate the risk of each outcome in fiscal year 2019 relative to 2009.

To estimate the independent association between the update to FTT and a patient’s risk of presenting to a trauma center, we performed an interrupted time series analysis using modified Poisson regression with an autoregressive correlation structure.^45^ To account for changes in population characteristics during the study, models were adjusted for patient age; sex; comorbid status; frailty; long-term home care use; nursing home residence; rural location; socioeconomic status; MOI; ISS; presence of a severe head, chest, or abdominal injury; and time of injury. To facilitate estimation of both the instantaneous association between FTT and the risk of presentation to a trauma center as well as the association between FTT and secular trends in trauma center presentation, unique variables were included in the model to represent the exposure period (preimplementation, peri-implementation, or postimplementation) as well as the time after each exposure interval.^45^ Consequently, the final model included 3 unique segments.^46^ To account for seasonality, the model was also adjusted for the season in which the injury occurred. Likewise, given that observations that occur closer together in time tend to be more similar than those that are further apart (ie, 2 patients injured in 2010 are more likely to have similar triage decisions than a patient injured in 2010 and another injured in 2018), we used autocorrelation function plots to evaluate the correlation between observations and various lag points. Based on this evaluation, a first order autoregressive correlation structure was used for the model. Similar models were used to estimate the risk of undertriage, restricted to severely injured patients, and overtriage, restricted to patients with minor injuries. Covariates related to presence of severe injuries were excluded from the model examining overtriage. After undergoing peer review, an additional post hoc analysis was performed. For this analysis, overtriage was examined from the perspective of the trauma center. We therefore evaluated the rate at which patients with minor injuries presented to a provincially designated lead trauma hospital relative to all patients that presented to these centers. Given that covariates related to presence of severe injuries and a patient’s ISS were part of the definition of the outcome, they were excluded from this model.

To examine the robustness of our results, we performed 5 sensitivity analyses (eAppendix 2 in Supplement 1). These analyses evaluated the effect of trauma center proximity, definition of implementation period, and definition of undertriage, on the association between FTT and triage patterns.

All analysis were performed using complete cases. Data were missing for socioeconomic status in 3417 encounters (1.2%), rural location in 123 encounters (<0.1%), MOI in 912 encounters (0.3%), and triage acuity in 115 encounters (<0.1%). The ISS could not be calculated in 11 995 encounters (4.3%) of cases.

Statistical analysis was performed using SAS software version 9.4 (SAS Institute). Standardized differences greater than 0.10 and 2-sided *P* < .05 were considered significant.^43^ Statistical analysis was conducted from October 2023 to October 2025.

## Results

During the 11-year study period, we identified 281 268 patients (mean [SD] age, 62.4 [22.5] years; 141 450 [50.3%] female; median [IQR] ISS, 4 [4-9]), including 37 174 (13.2%) with a severe injury. Most baseline characteristics were similar between patients injured before and after the implementation of new FTT guidelines (Table 2; eAppendix 3 in Supplement 1). However, patients injured after implementation were older than those injured prior to implementation.

**Table 2.** Baseline Characteristics of Adult Patients Presenting With Traumatic Injury to an Ontario Hospital Before and After Implementation of Updated Field Trauma Triage Guidelines

Characteristic	Patients, No. (%)			Standardized differences
	Overall (N = 281 286)	Preimplementation (n = 127 275)	Postimplementation (n = 130 680)	
Age, y				
Mean (SD)	62.4 (22.5)	60.4 (22.7)	64.1 (22.1)	0.16
≥65	142 980 (50.8)	59 862 (47.0)	70 938 (54.3)	0.15
Sex				
Female	141 450 (50.3)	63 152 (49.6)	66 350 (50.8)	0.02
Male	139 818 (49.7)	64 123 (50.4)	64 330 (49.2)	
Comorbidity level				
Low	110 340 (39.2)	52 390 (41.2)	48 748 (37.3)	0.08
Moderate	84 144 (29.9)	38 345 (30.1)	38 877 (29.7)	0.01
High	86 784 (30.9)	36 540 (28.7)	43 055 (32.9)	0.09
Frail	49 171 (17.5)	19 584 (15.4)	23 394 (19.4)	0.11
Long-term home care	29 004 (10.3)	12 224 (9.6)	14 306 (10.9)	0.04
Nursing home resident	23 423 (8.3)	8831 (6.9)	12 617 (9.7)	0.10
Rural	40 439 (14.4)	20 001 (15.7)	17 189 (13.2)	0.07
Marginalization score, mean (SD)	3.2 (0.8)	3.2 (0.8)	3.2 (0.8)	0.02
ISS, median (IQR)				
Median (IQR)	4 (4-9)	4 (4-9)	4 (4-9)	0.10
Category				
1-8	159 030 (56.5)	74 898 (58.8)	70 885 (54.2)	0.09
9-15	73 069 (26.0)	31 148 (24.5)	35 837 (27.4)	0.07
16-24	25 593 (9.1)	10 682 (8.4)	12 816 (9.8)	0.05
25-34	6289 (2.2)	2568 (2.0)	3228 (2.5)	0.03
35-75	5292 (1.9)	2305 (1.8)	2584 (2.0)	0.01
Mechanism of injury				
MVC	28 053 (10.0)	12 780 (10.0)	13 010 (10.0)	<0.01
Fall	190 611 (68.0)	84 148 (66.1)	90 351 (69.1)	0.06
Pedestrian or cyclist struck	14 555 (5.2)	6773 (5.3)	6580 (5.0)	0.01
Other blunt mechanism	34 637 (12.4)	17 581 (13.8)	14 331 (11.0)	0.09
Cut or pierce	10 677 (3.8)	5017 (3.9)	4865 (3.7)	0.01
GSW	1823 (0.7)	672 (0.7)	1016 (0.8)	0.03
Severe injury	37 174 (13.2)	15 555 (12.2)	18 628 (14.3)	0.06
ACS specified injury^a^	67 129 (23.9)	27 789 (21.8)	33 830 (25.9)	0.10
Triage score				
1 (highest acuity)	29 802 (10.6)	11 934 (9.4)	15 377 (11.8)	0.08
2	100 588 (35.8)	44 435 (34.9)	47 467 (36.3)	0.03
3	128 006 (45.5)	58 448 (45.9)	58 999 (45.1)	0.02
4	21 299 (7.6)	11 768 (9.2)	8038 (6.2)	0.12
5 (lowest acuity)	919 (0.3)	369 (0.3)	520 (0.4)	0.02
Time of presentation				
Weekday	107 327 (38.2)	48 342 (38.0)	49 950 (38.2)	<0.01
Evening or weekend	137 634 (48.9)	62 481 (49.1)	63 781 (48.8)	0.01
Night	36 307 (12.9)	16 452 (12.9)	16 949 (13.0)	<0.01
Season				
Spring	65 497 (23.3)	32 047 (25.2)	27 207 (20.8)	0.10
Summer	75 385 (26.8)	34 580 (27.2)	36 148 (27.7)	0.01
Fall	70 441 (25.0)	30 008 (23.6)	34 055 (26.1)	0.06
Winter	69 945 (24.9)	30 640 (24.1)	33 270 (25.5)	0.03

Abbreviations: ACS, American College of Surgeons; GSW, gunshot wound; ISS, Injury Severity Score; MVC, motor vehicle collision.

^a^ ACS specified injuries include injuries to the aorta, carotid, and/or vertebral vessels; injuries to the heart; multiple rib fractures; injuries to abdominal vasculature; open fractures with loss of distal pulse; open skull fractures; head injuries with a score on the Glassgow Coma Scale less than 14; spinal cord injuries; 2 or more vertebral column fractures; open fractures of a long bone; severe torso injuries with a comorbid condition; and grade IV liver lacerations.

Throughout the study, most patients initially presented to a nontrauma center (227 398 patients [80.9%]). Among 37 174 patients with a severe injury, 13 577 (36.5%) presented to a trauma center, while among 232 295 patients with a minor injury without high-risk features, 203 801 (87.7%) presented to a nontrauma center. Consequently, overall rates of undertriage and overtriage were 63.5% and 12.3%, respectively. Among 53 870 patients who presented directly to a trauma center, 28 494 (52.9%) had a minor injury without high-risk features.

### Temporal Trends in Trauma Triage

**Figure 1.**
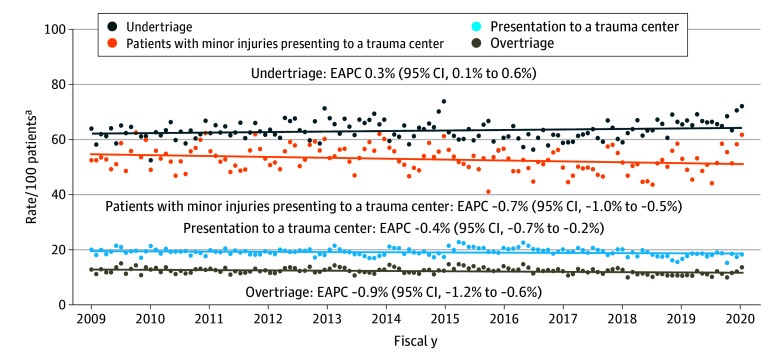
Temporal Trends in Unadjusted Rates of Transport to a Trauma Center, Undertriage, and Overtriage EAPC indicates estimated annual percent change. ^a^Rates of undertriage are relative to patients with severe injuries, rates of overtriage are relative to those with minor injuries, rates of patients with minor injuries presenting to trauma centers are relative to patients who presented to trauma centers.

Overall, the crude rate of trauma center presentation decreased by 0.4% (95% CI, 0.2%-0.7%) per year, with a patient’s likelihood of presenting to a trauma center being 7.5% lower in 2019 compared to 2009 (rate ratio [RR], 0.93; 95% CI, 0.89-0.96), (Figure 1). Likewise, rates of undertriage increased by 0.3% (95% CI, 0.1%-0.6%) per year, while rates of overtriage decreased by 0.9% (95% CI, 0.6%-1.2%) per year. Consequently, severely injured patients were 7.5% less likely and patients with minor injuries were 11.0% less likely to be transported to a trauma center in 2019 vs 2009 (severely injured: RR 0.93; 95% CI, 0.89-0.96; minor injury: RR, 0.89; 95% CI, 0.84-0.94). The rate at which patients with minor injuries presented directly to a trauma center also decreased throughout the study period (annual rate of decrease 0.7%; 95% CI, 0.5%-1.0%).

### Independent Association Between Updated FTT Criteria and Triage Rates

**Figure 2.**
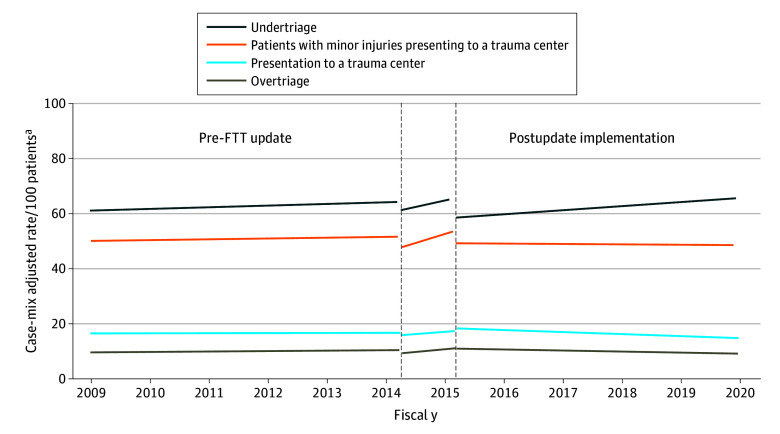
Case-Mix Adjusted Trends in Rates of Transport to a Trauma Center, Undertriage, and Overtriage FTT indicates field trauma triage ^a^Rates of undertriage are relative to patients with severe injuries, rates of overtriage are relative to those with minor injuries, rates of patients with minor injuries presenting to trauma centers are relative to patients who presented to trauma centers.

Interrupted time series analyses were used to evaluate the independent association between updated FTT guidelines and triage trends (Figure 2; eAppendix 4 in Supplement 1). Case mix–adjusted analyses demonstrated that during the 5 years prior to the implementation of FTT, the rate of patients presenting to a trauma center was constant (adjusted EAPC, 0.1%; 95% CI, −0.6% to 0.9%). Neither the announcement of FTT, the implementation period, or the final rollout were associated with a patient’s likelihood of presenting to a trauma center (Table 3). However, during the postimplementation period, the rate at which patients presented to a trauma center decreased relative to the baseline period (RR, 0.89; 95% CI, 0.81-0.98). Therefore, during the postimplementation period, case mix–adjusted rates of presentation to a trauma center decreased by 4.2% (95% CI, 3.4%-4.9%) per year.

**Table 3.** Interrupted Time Series Analysis Examining the Independent Association Between Updated FTT Guidelines and Triage Rates

Outcome	Rate ratio (95% CI)				
	Baseline period	Implementation period		Postimplementation period	
	Rate change, per 1-y	At announcement	Annual rate change vs baseline	At full rollout	Annual rate change vs baseline
Transport to a trauma center	1.00 (0.99-1.01)	0.96 (0.90-1.02)	1.07 (0.97-1.19)	1.02 (0.93-1.12)	0.89 (0.81-0.98)
Undertriage^a^	1.01 (1.00-1.02)	0.95 (0.89-1.01)	1.07 (0.97-1.18)	0.85 (0.77-0.94)	0.95 (0.86-1.05)
Population-level overtriage^b^	1.01 (1.00-1.03)	0.89 (0.82-0.97)	1.18 (1.02-1.36)	0.90 (0.79-1.04)	0.80 (0.70-0.93)
Trauma center-level overtriage^c^	1.00 (1.00-1.01)	0.92 (0.87-0.99)	1.13 (1.01-1.25)	0.86 (0.77-0.95)	0.88 (0.79-0.98)

^a^ Undertriage rates are relative to the number of patients with a severe injury.

^b^ Population-level overtriage rates are relative to the number of patients without a severe, critical, or life-threatening injury, who did not receive mechanical ventilation in the emergency department, did not receive blood within 24 hours of presentation, and survived greater than 24 hours.

^c^ Trauma center-level overtriage rates are relative to the total number of patients who initially presented to a provincially designated lead trauma hospital.

Given that decreases in the rate at which patients presented to trauma centers could reflect either increases in undertriage or decreases in overtriage, we examined the association between updated FTT guidelines and both undertriage and overtriage. Prior to the implementation of FTT, rates of undertriage were increasing by 1.0% (95% CI, 0.2%-1.8%) per year. Again, neither the announcement of FTT nor the implementation period were associated with a patient’s risk of undertriage (Table 3). However, the final rollout of FTT was associated with an instantaneous decrease in undertriage of 15.2% (RR, 0.85; 95% CI, 0.77 to 0.94). While the implementation of FTT was associated with a significant instantaneous drop in undertriage, after this drop, the annual increase in undertriage rates observed in the baseline period resumed during the postimplementation period. Consequently, even after the implementation of FTT guidelines, case mix–adjusted rates of undertriage continued to increase by 2.4% (95% CI, 1.7% to 3.2%) per year.

Among patients with minor injuries, prior to the implementation of FTT, rates of overtriage were increasing by 1.5% (95% CI, 0.5%-2.5%) per year. Unlike the association between the announcement of FTT guidelines and rates of trauma center transport or undertriage, the announcement was associated with an instantaneous reduction in overtriage of 10.8% (RR, 0.89; 95% CI, 0.82-0.97). The instantaneous outcome after the announcement was not sustained, and the implementation period was associated with an increase in overtriage rates relative to the baseline period. While the final rollout of FTT was not associated with an instantaneous change in the risk of overtriage, the postimplementation period had a significant decrease in overtriage rates relative to the baseline period (RR, 0.80; 95% CI, 0.70-0.93). Consequently, after FTT implementation case mix–adjusted rates of overtriage decreased by 3.8% (95% CI, 2.7%-5.0%) per year.

Examination of overtriage from the perspective of trauma centers demonstrated that prior to the implementation of FTT the rate at which patients with minor injuries presented directly to trauma centers was stable (adjusted EAPC, 0.4%; 95% CI, −0.3% to 1.2%). Similar to the association between the announcement of updated FTT guidelines and population-level rates of overtriage, the announcement was associated with an instantaneous reduction in the risk of patients with minor injuries presenting directly to trauma centers (RR, 0.92; 95% CI, 0.87-0.99). Likewise, the instantaneous effect of the announcement was not sustained, and the implementation period was associated with an increase in trauma center–level overtriage rates relative to the baseline period. However, the final rollout of the updated FTT guidelines was associated both with an instantaneous decrease in the likelihood that a patient with a minor injury would present directly to a trauma center (RR, 0.86; 95% CI, 0.77 to 0.95) and a decrease in the annual rate at which patients with minor injuries presented to trauma centers (RR, 0.88; 95% CI, 0.79 to 0.98). Despite these associations, given underlying temporal trends, after the instantaneous drop in the rate at which patients with minor injuries presented to trauma centers, the overall annual case mix–adjusted rate of trauma center–level overtriage remained stable during the postimplementation phase (adjusted EAPC, −0.3%; 95% CI, −1.2% to 0.5%).

### Sensitivity Analysis

Sensitivity analyses demonstrated that the association between updated FTT guidelines and triage rates was consistent regardless of trauma center proximity, definition of implementation period, or definition of undertriage (eAppendix 5 in Supplement 1). Specifically, FTT was associated with an instantaneous decrease in undertriage, and during the years after implementation, rates of overtriage decreased while rates of undertriage increased. Evaluation of the components of the expanded definition of undertriage suggested that the implementation of FTT guidelines had minimal association with undertriage rates among patients with specific clinical presentations, including those who required emergency ventilation or died early.

## Discussion

In this population-based study of the implementation of the 2015 update to Ontario FTT guidelines, we found that their implementation was associated with an instantaneous decrease in the rate of undertriage of 15% without a commensurate increase in overtriage. However, this reduction was not sustained. During the 5 years after the rollout of FTT, rates of undertriage increased by 2% annually. These findings suggest that while FTT was associated with decreasing undertriage without increasing overtriage, ongoing monitoring of triage practices is essential to ensure the benefits associated with FTT are maintained.

Our findings are consistent with the literature demonstrating that FTT can identify a significant proportion of severely injured patients.^7,8^ Moreover, our results address the concern that the implementation of FTT will result in unsustainable increases in overtriage.^47,48^ Contrary to these concerns, we demonstrated that updates to FTT guidelines were associated with decreasing rates of overtriage. However, we also found that despite an initial decrease in rates of undertriage, underlying trends in triage of severely injured patients remained unchanged. Consequently, rates of undertriage increased during the years after the implementation of FTT.

Current initiatives to reduce undertriage focus on refining FTT criteria.^6^ Our findings suggest that the ability of FTT criteria to identify severe injuries is not the primary driver of ongoing high rates of undertriage. While we did not have access to paramedic documentation to determine specific drivers of undertriage (ie, inappropriate application of FTT guidelines vs criteria not identifying patients with severe injuries), given the initial decrease it undertriage associated with the update to FTT, it is unlikely that failure of the guidelines to identify severely injured patients was the primary driver of subsequent increases in undertriage during the years after implementation. Rather, this finding highlights an alternative target for interventions to reduce undertriage: longitudinal feedback aimed at sustaining reductions in undertriage. It is possible that the structured training related to the implementation of FTT played a substantial role in the initial decrease in undertriage associated with these guidelines. Conversely, as clinicians became further removed from these sessions they may have returned to their previous triage practices, practices based on heuristics and pattern recognition.^17,49^ In other words, while clinicians still knew to transport severely injured patients to trauma centers, they reverted to the practice of transporting patients they perceived as severely injured rather than those flagged by guidelines. The challenge in translating clinical guidelines to the bedside is not unique to prehospital medicine. Evaluation of implementation strategies in cardiac care have demonstrated the key role of implementation sciences and benefit of audit and feedback mechanisms.^50,51^

### Limitations

There are important limitations to consider when interpreting the results of this study. First, due to data limitations, we could not determine method of transportation to hospital. It is likely that a portion of patients included in this study self-presented; therefore, FTT guidelines would have no effect on their hospital of presentation. As it is unlikely that methods of transport to hospital systematically varied during the study period, the associations demonstrated in study are likely robust to this limitation. Second, the identification of injury severity was based on administrative data. While the algorithm used in this study has been validated and is highly accurate, it is biased to underestimate injury severity.^36^ It is therefore likely that we underestimated the rate of undertriage. However, the same algorithm was applied across the study making it unlikely that underestimation of injury severity biased our results. Likewise, to limit the potential of inappropriately classifying patients with minor injuries as having severe injuries, we excluded all patients discharged home from the ED of the initial nontrauma center regardless of calculated ISS. We therefore biased our study in favor of the decision made by the treating clinicians and minimized estimated undertriage rates. Third, it is possible that residual confounding related to triage decisions remained. Administrative data does not include details related to hemodynamic status or level of consciousness. It is possible that some patients identified as overtriaged met physiologic criteria for trauma center presentation. To overcome this limitation, we extracted data related to ventilation and blood transfusion. These variables were used as surrogates for clinical status and used to limit the identification of patients as overtriaged. Consequently, it is unlikely that misclassification existed to a significant enough extent to alter the findings of our analysis. A fourth limitation is the inclusion of all injured patients regardless of location. Given the size of Ontario, 10% of the population does not have ready access to a provincially designated lead trauma hospital.^22^ FTT cannot alter the rate at which these patients are transported to a trauma center. To ensure inclusion of remote patients did not obscure the impact of FTT, we performed a sensitivity analysis restricted to patients whose destination hospital could most easily be impacted by EMS personnel. The results of this analysis remained consistent with the primary analysis. A fifth limitation is the scope of our study. We focused our analysis on the association between the update to FTT guidelines and triage accuracy. While our findings suggest that longitudinal audit and feedback mechanisms may be essential in maintaining improvements in triage accuracy associated with updates to FTT guidelines, guideline adherence is only a small piece of the puzzle. Rather, there are numerous potential causes of undertriage, including geographic barriers, implicit bias, and reliance on pattern recognition.^12,13,14,15,16,17^ Future research is essential in identifying strategies to ensure that all patients have optimal access to resources commensurate with their illness severity. Sixth, our analysis focused on the association between a 2015 update to the FTT guidelines based on recommendations from the 2011 National Expert Panel. In 2021, FTT guidelines were further updated with a focus on reducing undertriage.^6^ While ideally the implementation of these guidelines has further reduced undertriage within trauma systems, it is likely that the findings of our study still apply. That is, undertriage rates are likely to drop with initial implementation, but over time clinicians may revert to their baseline triage practices. Finally, because this study was centered around FTT guidelines, this study focuses only on primary triage (ie, triage by EMS in the field). This study does not assess the proportion of patients who eventually receive trauma center treatment thanks to interfacility transfer.

## Conclusions

The findings of this cohort study suggest that updates to FTT guidelines were associated with a transient decrease in undertriage without a commensurate increase in overtriage. However, their implementation was not associated with a difference in underlying trends related to the triage of severely injured patients. Consequently, during the years after implementation, undertriage rates gradually increased. These findings support the use of FTT to improve access to trauma care and highlight the need for ongoing monitoring of triage practices to ensure the benefits of novel interventions are preserved.

## Notes

Supplement 1. **eAppendix 1.** Datasets and Definitions **eAppendix 2.** Sensitivity Analyses **eAppendix 3.** Detailed Cohort Characteristics **eAppendix 4.** Details of the Interrupted Time Series Analyses Estimating the Independent Association Between of the Implementation of Updated Field Trauma Triage Guidelines on Triage Trends **eAppendix 5.** Sensitivity Analyses

Supplement 2.
Data Sharing Statement
