# Supplementary material for: Triage Accuracy and the 2015 Field Trauma Triage Criteria Update
Source: JAMA Netw Open. 2026 Jan 5;9(1):e2552092. doi: 10.1001/jamanetworkopen.2025.52092 (PMC12771241; doi:10.1001/jamanetworkopen.2025.52092)
Supplement: Supplement 1. — eAppendix 1. Datasets and Definitions eAppendix 2. Sensitivity Analyses eAppendix 3. Detailed Cohort Characteristics eAppendix 4. Details of the Interrupted Time Series Analyses Estimating the Independent Association Between of the Implementation of Updated Field Trauma Triage Guidelines on Triage Trends eAppendix 5. Sensitivity Analyses [file jamanetwopen-e2552092-s001.pdf]

Supplemental Online Content

Tillmann BW, Nathens AB, Guttman MP, et al. Triage Accuracy and the 2015 field trauma triage criteria update. *JAMA Netw Open*. 2026;9(1):e2552092.
doi:10.1001/jamanetworkopen.2025.52092

eAppendix 1. Datasets and Definitions
eAppendix 2. Sensitivity Analyses
eAppendix 3. Detailed Cohort Characteristics
eAppendix 4. Details of the Interrupted Time Series Analyses Estimating the Independent Association Between of the Implementation of Updated Field Trauma Triage Guidelines on Triage Trends
eAppendix 5. Sensitivity Analyses

This supplemental material has been provided by the authors to give readers additional information about their work.

© 2025 Tillmann BW et al. *JAMA Network Open*.

eAppendix A. Methods – Datasets and definitions

Data were obtained from several administrative databases in Ontario, Canada. All databases aside from Ornge are held at ICES

Table 1: Details of administrative datasets

Database name	Description	Data derived from each database
National Ambulatory Care Reporting System (NACRS)	Contains information on all emergency department visits in Ontario	• Identification of patients for study inclusion<br>• Identification of triage status<br>• Patient baseline characteristics<br>• Identification of triage status
Discharge Abstract Database (DAD)	Contains information on all acute care hospitalizations in Ontario	• Identification of triage status<br>• Patient baseline characteristics<br>• Identification of comorbid conditions
Registered Persons Database (RPDB)	Contains demographic information on all residents of Ontario with a health card	• Patient demographic information
Ontario Health Insurance Plan Claims database (OHIP)	Captures physician services and encounters through billing claims	• Patient baseline characteristics
Ontario Marginalization Index (ONMARG)	Contains information on all residents on Ontario pertaining to their social economic status	• Patient socioeconomic information
Home Care Database (HCD)	Contains information on all publicly funded home care services in Ontario	• Identification of patients receiving homecare
Client Profile Database (CPRO)	Tracks applications to publicly funded nursing home facilities	• Identification of patients on nursing home waitlist
Continuing Care Reporting System (CCRS)	Contains demographic and clinical information on patients residing in publicly funded nursing home facilities	• Identification of patients residing in nursing home

© 2025 Tillmann BW et al. *JAMA Network Open*.

Table 2: Definition of covariates

Variable	Definition	Sources	Type	Analysis format
**Patient and injury characteristics**				
Age	Age at time of injury	RPDB	Continuous	Continuous variable
Sex	Biologic sex	RPDB	Dichotomous	Male/Female
Comorbidity level	Measured using the Johns Hopkins Adjusted Clinical Groups® (ACG) System Version 10 based on health services use with a 24-month look-back window prior to the date of injury whereby the 32 aggregated diagnosis groups (ADG) are summed to create a total score (1)	DAD<br>OHIP	Categorical	Low (0 – 4)<br>Medium (5 – 9)<br>High (≥10)
Presence of frailty	Identified based on the presence of one or more diagnoses from 12 clusters of frailty-related conditions specified by the ACG system during the two years prior to their injury (2)	DAD<br>OHIP	Dichotomous	Yes/No
Chronic homecare	Defined as the receipt of publicly funded, long-term in-home supportive care within 90 days preceding the injury	HCD	Dichotomous	Yes/No
Nursing home residence	Defined as a patient who either lived in or had been accepted to a publicly funded nursing home.<br>As acceptance to nursing home indicated a patient's functional status had declined to a point where they were no longer safe to live independently, these patients were also identified as nursing home residents	CCRS<br>CPRO	Dichotomous	Yes/No
Rural location	Determined using postal code of residence and the "rural and small town" definition used by Statistics Canada (3)	RPDB	Dichotomous	Urban/Rural
Socioeconomic status	Determined using postal code of residence and the Canadian Marginalization Index (4)	ONMARG	Summary score is continuous, individual subscales are categorical	Score on individual subscales<br>• 1 (Lowest)<br>• 2<br>• 3<br>• 4

© 2025 Tillmann BW et al. *JAMA Network Open*.

Variable	Definition	Sources	Type	Analysis format
				• 5 (Highest)
ISS	A validated algorithm was used to calculate the ISS for each patient based on the diagnostic codes recorded in their first hospital admission or, if not admitted, their last ED encounter (5)	DAD<br>NACRS	Categorical	1 – 8<br>9 – 15<br>16 – 24<br>25 – 34<br>35 – 75
Presence of a severe head injury	A validated algorithm was used to calculate the AIS severity score for each injury based on the diagnostic codes recorded in their first hospital admission or, if not admitted, their last ED encounter (5). If the AIS score for the body region head was ≥ 3 then they were identified as having a severe head injury.	NACRS<br>DAD	Dichotomous	Yes/No
Presence of a severe chest injury	A validated algorithm was used to calculate the AIS severity score for each injury based on the diagnostic codes recorded in their first hospital admission or, if not admitted, their last ED encounter (5). If the AIS score for the body region chest was ≥ 3 then they were identified as having a severe chest injury.	NACRS<br>DAD	Dichotomous	Yes/No
Presence of a severe abdominal injury	A validated algorithm was used to calculate the AIS severity score for each injury based on the diagnostic codes recorded in their first hospital admission or, if not admitted, their last ED encounter (5). If the AIS score for the body region abdomen was ≥ 3 then they were identified as having a severe abdominal injury.	NACRS<br>DAD	Dichotomous	Yes/No
MOI	The mechanism of injury was determined based on the external cause codes recorded in their first hospital admission or, if not admitted, their last ED encounter as recommended by the Centers for Disease Control and Prevention (6).	DAD<br>NACRS	Categorical	MVC<br>Fall<br>Pedestrian/cyclist struck<br>Other blunt mechanism<br>Cut/pierce<br>GSW
Date and time of presentation	Date and time of presentation at the non-trauma centre	NACRS	Categorical	Year<br>Day of the week

© 2025 Tillmann BW et al. *JAMA Network Open*.

Variable	Definition	Sources	Type	Analysis format
				Time period<br>• 07:00 – 17:00<br>• 17:01 – 23:59<br>• 00:00 – 06:59<br>Season<br>• Spring<br>• Summer<br>• Fall<br>• Winter
Triage acuity	Triage score assigned using the Canadian Triage Acuity Scale (7)	NACRS	Categorical	1 (Highest)<br>2<br>3<br>4<br>5 (Lowest)

ISS = Injury Severity Score; MOI = Mechanism of Injury; ACG = Adjusted Clinical Groups®; ADG = Aggregated diagnosis groups; ED = Emergency department; AIS = Abbreviated injury scale; RPDB = Registered Persons Database; DAD = Discharge Abstract Database; OHIP = Ontario Health Insurance Plan Claims database; HCD = Home Care Database; CCRS = Continuing Care Reporting System; CPRO = Client Profile Database; ONMARG = Ontario Marginalization Index; NACRS = National Ambulatory Care Reporting System; MVC = Motor vehicle collision; GSW = Gunshot wound;

1) Reid RJ, MacWilliam L, Verhulst L, Roos N, Atkinson M. Performance of the ACG case-mix system in two Canadian provinces. *Med Care*. Jan 2001;39(1):86-99.
2) Ho MM, Camacho X, Gruneir A, Bronskill SE. Overview of Cohorts, *In: Health System Use by Frail Ontario Seniors: An In-Depth Examination of Four Vulnerable Cohorts*. Institute for Clinical Evaluative Sciences; 2011.
3) du Plessis V, Beshiri R, Bollman RD, Clemenson H. Definitions of rural. In: Statistics Canada editor. *Rural and Small Town Canada Analysis Bulletin*. Ottawa, Ontario: Statistics Canada; 2001.
4) Matheson FI, Dunn JR, Smith KL, Moineddin R, Glazier RH. Development of the Canadian Marginalization Index: a new tool for the study of inequality. *Can J Public Health*. Apr 2012;103(8 Suppl 2):S12-6.
5) Tillmann BW, Guttman MP, Thakore J, et al. Internal and external validation of an updated ICD-10-CA to AIS-2005 Update 2008 algorithm. *J Trauma Acute Care Surg*. Jul 05 2023;doi:10.1097/TA.0000000000004052
6) Fingerhut LA, Warner M. The ICD–10 injury mortality diagnosis matrix. Inj Prev 12(1):24–9. 2006. Available from: https://injuryprevention-bmj-com.myaccess.library.utoronto.ca/content/12/1/24
7) J Murray M. The Canadian Triage and Acuity Scale: A Canadian perspective on emergency department triage. *Emerg Med (Fremantle)*. Feb 2003;15(1):6-10.

© 2025 Tillmann BW et al. *JAMA Network Open*.

Table 3: Injuries defined as critical or life-threatening by the American College of Surgeons

Injury	ICD-10-CA Code
Injury to aorta, carotid, and/or vertebral vessels	S15.0 – S12.9 and S25.0 – S25.9
Injury to the heart	S26.0 – S26.9
Multiple rib fractures	S22.41, S22.49, and S22.5
Injury to abdominal vasculature	S35.0 – S35.5
Open fracture with loss of distal pulse	S48.0 – S48.9, S58.0 – S58.9, S68.4, S78.0 – S78.9, S88.0 – S88.9, S98.0, T05.0 – T05.6, and T05.9
Open skull fracture	S02.001, S02.101, S02.411, S02.421, S02.431, S02.441, S02.701, S02.891, and S02.901
Head injury with a GCS <14	AIS score in the body region head ≥3
Any spinal cord injury or more than 1 vertebral column fracture	S14.0, S14.1, S14.2, S14.5, S24.0, S24.1, S24.2, S24.4, S34.0, S34.1, S34.2, S34.5, T06.0, T06.1, S12.7, S22.1, S32.7, and any combination of the following S12.0, S12.1, S12.2, S12.9, S22.0, and S32.0
Open fracture of a long bone	S42.2_1, S42.3_1, S42.4_1, S52.0_1, S52.1_1, S52.201, S52.301, S52.401, S52.701, S52.801, S52.901, T02.21, T02.41, S72.0_1, S72.1_1, S72.201, S72.301, S72.4_1, S72.701, S72.801, S72.901, S82.101, S82.201, S82.301, S82.701, S82.901, T02.31, T02.51, and T02.61,
Severe torso injury with a comorbid condition	S27.41, S27.48, S27.51, S27.58, S27.71, S27.78, S27.8, S27.9, T04.1, T04.7, T05.8, T06.5, S28, S36.02, S36.03, S36.04, S36.12, S36.13, S36.14, S36.18, S36.22, S36.23, S36.24, S36.31, S36.41, S36.42, S36.46, S36.51, S36.61, S36.71, S36.78, S36.81, S36.91, S37.02, S37.03, S37.11, S37.21, S37.61, S37.71, S37.81, S37.91, S38.2, S38.3, and S39.6<br><br>Comorbid conditions were identified using the Charlson Comorbidity Index Deyo modification
Grade IV liver laceration	S36.13, S36.14

See *Mohan D, Rosengart MR, Farris C, Cohen E, Angus DC, Barnato AE. Assessing the feasibility of the American College of Surgeons' benchmarks for the triage of trauma patients. Arch Surg. Jul 2011;146(7):786-92. doi:10.1001/archsurg.2011.43* and *Rotondo MF, Cribari C, Smith RS. Resources for Optimal Care of the Injured Patient 2014 (6th Edition). Committee on Trauma American College of Surgeons; 2014.*

© 2025 Tillmann BW et al. *JAMA Network Open*.

eAppendix B. **Methods – Sensitivity Analyses**

To examine the robustness of our results we performed five sensitivity analyses. In the first analysis we restricted the cohort to patients for who lived within 50 miles of a provincially designated lead trauma hospital, yet their nearest hospital was a non-trauma center. The goal of this analysis was to ensure the impact of FTT was not masked by patients who were either too far from a trauma center to be directly transported to one, or who lived so close to trauma center that they would be transported to one regardless of injury severity. In the next two sensitivity analyses, we condensed the implementation of FTT to a single time point, where the one-year announcement period was removed from the analysis. In the first model, we categorized all patients having been injured on or after July 1, 2014, as being treated in the post-FTT period. Conversely, in the second sensitivity analysis, we categorized all patients treated up until May 31, 2015, as being treated in the pre-FTT period. In the fourth sensitivity analysis we categorized all patients with high-risk injuries who were not transported to a trauma center as undertriaged. This analysis was undertaken to determine if a more inclusive definition of undertriage modified the impact of FTT on undertriage rates. In the final analysis, we evaluated the impact of FTT on each element of the extended definition of undertriage (ISS ≥ 16, death within 24hrs, ACS specific injury, ventilation, blood transfusion).

© 2025 Tillmann BW et al. *JAMA Network Open*.

eAppendix C. **Results – Detailed cohort characteristics**

Figure 1. Cohort Creation

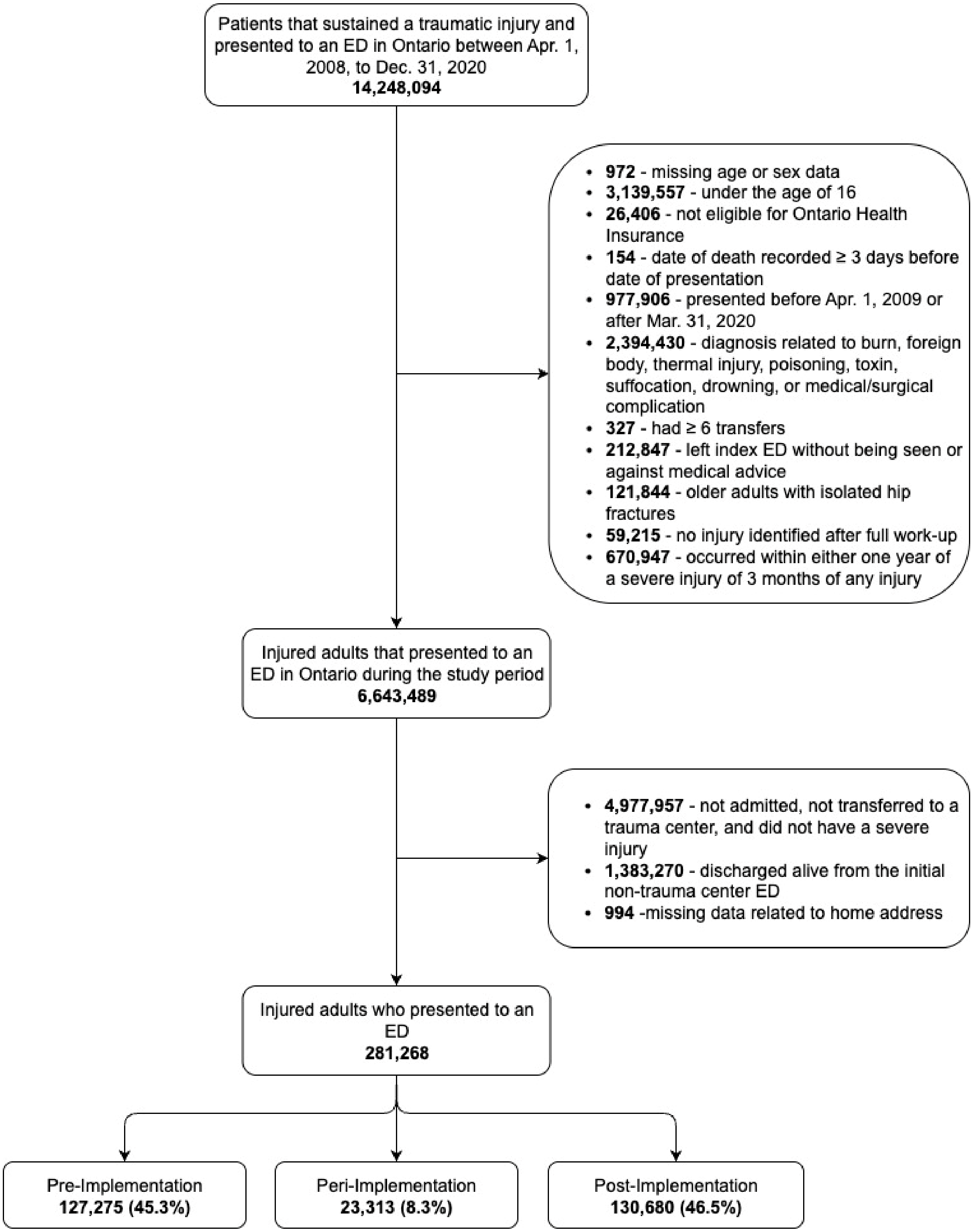

© 2025 Tillmann BW et al. *JAMA Network Open*.

Table 1. Additional baseline characteristics

	All patients (n=281,286)	Pre-Implementation (n=127,275)	Post-Implementation (n=130,680)	Standardized differences
Income quintile, n (%)				
1 (lowest)	67,490 (24.0)	28,700 (22.5)	32,950 (25.2)	0.06
2	58,957 (21.0)	25,970 (20.4)	27,964 (21.4)	0.02
3	53,556 (19.0)	24,503 (19.3)	24,622 (18.8)	0.01
4	50,913 (18.1)	24,159 (19.0)	22,731 (17.4)	0.04
5 (highest)	49,393 (17.6)	23,183 (18.2)	22,243 (17.0)	0.03
Housing and dwellings quintile, n (%)				
1 (lowest)	38,658 (13.7)	17,011 (13.4)	18,390 (14.1)	0.02
2	46,609 (16.6)	21,120 (16.6)	21,648 (16.6)	<0.01
3	51,579 (18.3)	23,458 (18.4)	23,799 (18.2)	0.01
4	59,491 (21.2)	27,381 (21.5)	27,256 (20.9)	0.02
5 (highest)	81,514 (29.0)	36,794 (28.9)	37,981 (29.1)	<0.01
Material resources quintile, n (%)				
1 (lowest)	49,577 (17.6)	20,867 (16.4)	24,362 (18.6)	0.06
2	52,110 (18.5)	23,273 (18.3)	24,483 (18.7)	0.01
3	53,231 (18.9)	24,646 (19.4)	24,260 (18.6)	0.02
4	56,419 (20.1)	25,764 (20.2)	25,926 (19.8)	0.01
5 (highest)	66,514 (23.7)	31,214 (24.5)	30,043 (23.0)	0.04
Age and labour force quintile, n (%)				
1 (lowest)	44,240 (15.7)	19,434 (15.3)	21,219 (16.2)	0.02
2	47,697 (17.0)	21,141 (16.6)	22,518 (17.2)	0.02
3	49,430 (17.6)	22,867 (18.0)	22,489 (17.2)	0.02
4	54,194 (19.3)	25,188 (19.8)	24,658 (18.9)	0.02
5 (highest)	82,290 (29.3)	37,134 (29.2)	38,280 (29.3)	<0.01
Racialized and newcomer population quintile, n (%)				
1 (lowest)	63,319 (22.5)	29,469 (23.2)	28,600 (21.9)	0.03
2	59,699 (21.2)	27,713 (21.8)	27,190 (20.8)	0.02
3	52,698 (18.7)	23,903 (18.8)	24,365 (18.6)	<0.01
4	50,696 (18.0)	22,733 (17.9)	23,737 (18.2)	0.01
5 (highest)	51,439 (18.3)	21,946 (17.2)	25,182 (19.3)	0.05

© 2025 Tillmann BW et al. *JAMA Network Open*.

eAppendix D. **Results – Details of the interrupted time series analyses estimating the independent association between of the implementation of updated field trauma triage (FTT) guidelines on triage trends**

Multivariable modified Poisson regression was used to estimate the independent association between the implementation of updated FTT guidelines and triage rates adjusting for differences in patient characteristics during the study period

Table 1. Case-mix adjusted association between the implementation of updated FTT guidelines and presentation to a trauma center

	Relative risk (95% CI)	p-value
Time pre-implementation (risk associated with one-year increase)	1.00 (0.99 – 1.01)	0.71
Instantaneous impact of announcement	0.96 (0.90 – 1.02)	0.15
Time peri-implementation (risk associated with one-year increase)	1.07 (0.97 – 1.19)	0.15
Instantaneous impact of full rollout	1.02 (0.93 – 1.12)	0.66
Time post-implementation (risk associated with one-year increase)	0.89 (0.81 – 0.98)	0.02
Case-mix adjusted risk associated with one-year increase in time during the post-implementation period	0.96 (0.95 – 0.97)	<0.001
Age (risk associated with one year increase in age)	0.99 (0.99 – 0.99)	<0.001
Sex		
Female	REF	–
Male	1.12 (1.10 – 1.14)	<0.001
Rural residence		
No	REF	–
Yes	0.40 (0.39 – 0.42)	<0.001
Comorbidity level		
Low	REF	–
Moderate	1.05 (1.04 – 1.07)	<0.001
High	1.07 (1.05 – 1.10)	<0.001
Frail		
No	REF	–
Yes	1.03 (1.00 – 1.06)	0.05
Chronic home care		
No	REF	–
Yes	1.01 (0.98 – 1.04)	0.66
Nursing home resident		
No	REF	–
Yes	0.94 (0.90 – 0.98)	0.001
Income quintile		
1 (lowest)	0.94 (0.91 – 0.97)	<0.001
2	0.99 (0.96 – 1.01)	0.30
3	REF	–
4	0.94 (0.92 – 0.97)	<0.001
5 (highest)	0.98 (0.95 – 1.01)	0.13
Households and dwellings quintile		
1 (lowest marginalization)	0.85 (0.83 – 0.88)	<0.001
2	0.92 (0.89 – 0.94)	<0.001
3	REF	–

© 2025 Tillmann BW et al. *JAMA Network Open*.

4	1.04 (1.02 – 1.07)	<0.001
5 (highest marginalization)	1.37 (1.34 – 1.40)	<0.001
Material resources quintile		
1 (lowest marginalization)	1.18 (1.15 – 1.21)	<0.001
2	1.09 (1.06 – 1.12)	<0.001
3	REF	–
4	1.02 (0.99 – 1.05)	0.16
5 (highest marginalization)	1.16 (1.13 – 1.19)	<0.001
Age and labour force quintile		
1 (lowest marginalization)	0.89 (0.87 – 0.92)	<0.001
2	0.97 (0.95 – 0.99)	0.01
3	REF	–
4	0.95 (0.93 – 0.98)	0.001
5 (highest marginalization)	0.80 (0.78 – 0.82)	<0.001
Racialized and newcomer populations quintile		
1 (lowest marginalization)	0.97 (0.94 – 0.99)	0.01
2	1.01 (0.98 – 1.03)	0.67
3	REF	–
4	1.00 (0.98 – 1.03)	0.86
5 (highest marginalization)	0.84 (0.82 – 0.86)	<0.001
ISS category		
1 – 8	REF	–
9 – 15	1.08 (1.06 – 1.11)	<0.001
16 – 24	1.65 (1.60 – 1.70)	<0.001
25 – 34	1.98 (1.90 – 2.07)	<0.001
35 – 75	2.02 (1.94 – 2.11)	<0.001
Mechanism of injury		
Fall	0.62 (0.61 – 0.63)	<0.001
MVC	REF	–
Pedestrian/cyclist struck	0.96 (0.93 – 0.99)	0.003
Other blunt	0.71 (0.69 – 0.73)	<0.001
Cut/pierce	1.11 (1.07 – 1.14)	<0.001
GSW	1.44 (1.37 – 1.50)	<0.001
Severe head injury		
No	REF	–
Yes	1.12 (1.09 – 1.15)	<0.001
Severe chest injury		
No	REF	–
Yes	0.99 (0.96 – 1.02)	0.46
Severe abdominal injury		
No	REF	–
Yes	1.03 (0.98 – 1.08)	0.26
Time of presentation		
Day	REF	–
Evening/weekend	1.00 (0.99 – 1.02)	0.67
Night	1.10 (1.08 – 1.13)	<0.001
Season		
Spring	1.03 (1.01 – 1.06)	0.003
Summer	REF	–
Fall	1.00 (0.98 – 1.02)	0.96
Winter	1.01 (0.99 – 1.04)	0.20

CI = Confidence Interval; ISS = Injury Severity Score; MVC = Motor Vehicle Collision; GSW = Gunshot wound

© 2025 Tillmann BW et al. *JAMA Network Open*.

Table 2. Case-mix adjusted association between the implementation of updated FTT guidelines and undertriage

	Relative risk (95% CI)	p-value
Time pre-implementation (risk associated with one-year increase)	1.01 (1.00 – 1.02)	0.01
Instantaneous impact of announcement	0.95 (0.89 – 1.01)	0.07
Time peri-implementation (risk associated with one-year increase)	1.07 (0.97 – 1.18)	0.18
Instantaneous impact of full rollout	0.85 (0.77 – 0.94)	0.001
Time post-implementation (risk associated with one-year increase)	0.95 (0.86 – 1.05)	0.28
Case-mix adjusted risk associated with one-year increase in time during the post-implementation period	1.02 (1.02 – 1.03)	<0.001
Age (risk associated with one year increase in age)	1.004 (1.003 – 1.004)	<0.001
Sex		
Female	REF	–
Male	0.97 (0.95 – 0.99)	<0.001
Rural residence		
No	REF	–
Yes	1.22 (1.20 – 1.25)	<0.001
Comorbidity level		
Low	REF	–
Moderate	0.97 (0.95 – 0.99)	0.002
High	0.98 (0.96 – 1.00)	0.05
Frail		
No	REF	–
Yes	0.99 (0.97 – 1.02)	0.67
Chronic home care		
No	REF	–
Yes	1.02 (0.99 – 1.05)	0.21
Nursing home resident		
No	REF	–
Yes	1.09 (1.06 – 1.13)	<0.001
Income quintile		
1 (lowest)	1.03 (1.00 – 1.06)	0.03
2	1.02 (0.99 – 1.04)	0.15
3	REF	–
4	1.01 (0.99 – 1.04)	0.24
5 (highest)	1.03 (1.00 – 1.05)	0.05
Households and dwellings quintile		
1 (lowest marginalization)	1.06 (1.03 – 1.09)	<0.001
2	1.02 (1.00 – 1.05)	0.07
3	REF	–
4	1.00 (0.98 – 1.02)	0.92
5 (highest marginalization)	0.89 (0.87 – 0.91)	<0.001
Material resources quintile		
1 (lowest marginalization)	0.92 (0.90 – 0.94)	<0.001
2	0.97 (0.95 – 0.99)	0.007
3	REF	–
4	1.00 (0.98 – 1.03)	0.88
5 (highest marginalization)	0.94 (0.92 – 0.97)	<0.001

© 2025 Tillmann BW et al. *JAMA Network Open*.

Age and labour force quintile		
1 (lowest marginalization)	1.06 (1.03 – 1.09)	<0.001
2	1.01 (0.98 – 1.04)	0.52
3	REF	–
4	1.02 (0.99 – 1.04)	0.14
5 (highest marginalization)	1.07 (1.05 – 1.10)	<0.001
Racialized and newcomer populations quintile		
1 (lowest marginalization)	1.04 (1.02 – 1.07)	<0.001
2	1.02 (1.00 – 1.05)	0.07
3	REF	–
4	0.93 (0.91 – 0.96)	<0.001
5 (highest marginalization)	0.90 (0.87 – 0.92)	<0.001
ISS category		
16 – 24	REF	–
25 – 34	0.87 (0.85 – 0.89)	<0.001
35 – 75	0.86 (0.84 – 0.89)	<0.001
Mechanism of injury		
Fall	1.17 (1.14 – 1.19)	<0.001
MVC	REF	–
Pedestrian/cyclist struck	0.90 (0.87 – 0.93)	0.003
Other blunt	1.15 (1.12 – 1.18)	<0.001
Cut/pierce	0.78 (0.73 – 0.82)	<0.001
GSW	0.54 (0.47 – 0.61)	<0.001
Severe head injury		
No	REF	–
Yes	0.96 (0.94 – 0.98)	<0.001
Severe chest injury		
No	REF	–
Yes	1.00 (0.98 – 1.02)	0.80
Severe abdominal injury		
No	REF	–
Yes	1.01 (0.96 – 1.05)	0.80
Time of presentation		
Day	REF	–
Evening/weekend	1.01 (0.99 – 1.02)	0.34
Night	0.99 (0.96 – 1.01)	0.34
Season		
Spring	0.98 (0.96 – 1.00)	0.04
Summer	REF	–
Fall	0.99 (0.97 – 1.01)	0.20
Winter	0.99 (0.96 – 1.01)	0.17

CI = Confidence Interval; ISS = Injury Severity Score; MVC = Motor Vehicle Collision; GSW = Gunshot wound

© 2025 Tillmann BW et al. *JAMA Network Open*.

Table 3. Case-mix adjusted association between the implementation of updated FTT guidelines and overtriage

	Relative risk (95% CI)	p-value
Time pre-implementation (risk associated with one-year increase)	1.01 (1.00 – 1.03)	0.005
Instantaneous impact of announcement	0.89 (0.82 – 0.97)	0.009
Time peri-implementation (risk associated with one-year increase)	1.18 (1.02 – 1.36)	0.02
Instantaneous impact of full rollout	0.90 (0.79 – 1.04)	0.15
Time post-implementation (risk associated with one-year increase)	0.80 (0.70 – 0.93)	0.003
Case-mix adjusted risk associated with one-year increase in time during the post-implementation period	0.96 (0.95 – 0.97)	<0.001
Age (risk associated with one year increase in age)	0.99 (0.99 – 0.99)	<0.001
Sex		
Female	REF	–
Male	1.07 (1.05 – 1.10)	<0.001
Rural residence		
No	REF	–
Yes	0.32 (0.3 – 0.34)	<0.001
Comorbidity level		
Low	REF	–
Moderate	1.03 (1.01 – 1.06)	0.02
High	1.04 (1.01 – 1.07)	0.02
Frail		
No	REF	–
Yes	1.03 (0.99 – 1.07)	0.09
Chronic home care		
No	REF	–
Yes	1.03 (0.99 – 1.07)	0.15
Nursing home resident		
No	REF	–
Yes	1.00 (0.95 – 1.05)	0.88
Income quintile		
1 (lowest)	0.96 (0.92 – 1.00)	0.03
2	0.99 (0.95 – 1.03)	0.59
3	REF	–
4	0.93 (0.90 – 0.97)	<0.001
5 (highest)	1.01 (0.97 – 1.05)	0.73
Households and dwellings quintile		
1 (lowest marginalization)	0.78 (0.75 – 0.82)	<0.001
2	0.86 (0.83 – 0.90)	<0.001
3	REF	–
4	1.08 (1.04 – 1.12)	<0.001
5 (highest marginalization)	1.50 (1.45 – 1.56)	<0.001
Material resources quintile		
1 (lowest marginalization)	1.19 (1.14 – 1.24)	<0.001
2	1.10 (1.06 – 1.14)	<0.001
3	REF	–
4	1.02 (0.98 – 1.06)	0.28

© 2025 Tillmann BW et al. *JAMA Network Open*.

5 (highest marginalization)	1.18 (1.14 – 1.23)	<0.001
Age and labour force quintile		
1 (lowest marginalization)	0.87 (0.84 – 0.90)	<0.001
2	0.97 (0.94 – 1.00)	0.07
3	REF	–
4	0.94 (0.90 – 0.97)	<0.001
5 (highest marginalization)	0.75 (0.72 – 0.78)	<0.001
Racialized and newcomer populations quintile		
1 (lowest marginalization)	1.00 (0.96 – 1.03)	0.79
2	1.02 (0.99 – 1.06)	0.15
3	REF	–
4	0.98 (0.95 – 1.02)	0.36
5 (highest marginalization)	0.72 (0.70 – 0.75)	<0.001
ISS category		
1 – 8	REF	–
9 – 15	0.54 (0.53 – 0.56)	<0.001
Mechanism of injury		
Fall	0.61 (0.59 – 0.63)	<0.001
MVC	REF	–
Pedestrian/cyclist struck	0.90 (0.85 – 0.95)	<0.001
Other blunt	0.70 (0.67 – 0.73)	<0.001
Cut/pierce	1.06 (1.01 – 1.11)	0.02
GSW	1.31 (1.18 – 1.46)	<0.001
Time of presentation		
Day	REF	–
Evening/weekend	1.02 (0.99 – 1.04)	0.20
Night	1.14 (1.10 – 1.18)	<0.001
Season		
Spring	1.02 (0.99 – 1.05)	0.23
Summer	REF	–
Fall	0.98 (0.95 – 1.01)	0.25
Winter	1.02 (0.99 – 1.05)	0.12

CI = Confidence Interval; ISS = Injury Severity Score; MVC = Motor Vehicle Collision; GSW = Gunshot wound

© 2025 Tillmann BW et al. *JAMA Network Open*.

Table 4. Case-mix adjusted association between the implementation of updated FTT guidelines and likelihood that a patient with minor injuries presented to trauma centers

	Relative risk (95% CI)	p-value
Time pre-implementation (risk associated with one-year increase)	1.00 (1.00 – 1.01)	0.29
Instantaneous impact of announcement	0.92 (0.87 – 0.99)	0.02
Time peri-implementation (risk associated with one-year increase)	1.13 (1.01 – 1.25)	0.03
Instantaneous impact of full rollout	0.86 (0.77 – 0.95)	0.003
Time post-implementation (risk associated with one-year increase)	0.88 (0.79 – 0.98)	0.02
Case-mix adjusted risk associated with one-year increase in time during the post-implementation period	1.00 (0.99 – 1.01)	0.43
Age (risk associated with one year increase in age)	0.997 (0.997 – 0.997)	<0.001
Sex		
Female	REF	
Male	0.78 (0.77 – 0.79)	<0.001
Rural residence		
No	REF	
Yes	0.87 (0.84 – 0.91)	<0.001
Comorbidity level		
Low	REF	
Moderate	0.97 (0.96 – 0.99)	0.01
High	0.97 (0.95 – 0.99)	0.008
Frail		
No	REF	
Yes	0.97 (0.95 – 1.00)	0.02
Chronic home care		
No	REF	
Yes	1.08 (1.05 – 1.11)	<0.001
Nursing home resident		
No	REF	
Yes	1.07 (1.03 – 1.10)	<0.001
Income quintile		
1 (lowest)	1.02 (0.98 – 1.05)	0.34
2	1.01 (0.99 – 1.04)	0.29
3	REF	
4	1.00 (0.98 – 1.03)	0.75
5 (highest)	1.03 (1.00 – 1.06)	0.04
Households and dwellings quintile		
1 (lowest marginalization)	0.95 (0.92 – 0.98)	0.003
2	0.95 (0.92 – 0.98)	0.002
3	REF	
4	1.03 (1.00 – 1.06)	0.02
5 (highest marginalization)	1.07 (1.04 – 1.09)	<0.001
Material resources quintile		
1 (lowest marginalization)	1.00 (0.97 – 1.03)	0.95
2	1.00 (0.97 – 1.02)	0.72
3	REF	
4	1.00 (0.98 – 1.03)	0.84
5 (highest marginalization)	1.02 (0.99 – 1.06)	0.12
Age and labour force quintile		

© 2025 Tillmann BW et al. *JAMA Network Open*.

1 (lowest marginalization)	1.00 (0.97 – 1.03)	0.84
2	1.00 (0.98 – 1.03)	0.72
3	REF	
4	0.99 (0.97 – 1.02)	0.59
5 (highest marginalization)	0.98 (0.95 – 1.00)	0.05
Racialized and newcomer populations quintile		
1 (lowest marginalization)	1.04 (1.02 – 1.07)	0.001
2	1.03 (1.01 – 1.06)	0.009
3	REF	
4	0.98 (0.96 – 1.01)	0.21
5 (highest marginalization)	0.87 (0.85 – 0.90)	<0.001
Mechanism of injury		
Fall	1.66 (1.61 – 1.71)	<0.001
MVC	REF	
Pedestrian/cyclist struck	1.03 (0.98 – 1.08)	0.19
Other blunt	1.61 (1.56 – 1.67)	<0.001
Cut/pierce	1.48 (1.42 – 1.54)	<0.001
GSW	0.75 (0.68 – 0.84)	<0.001
Time of presentation		
Day	REF	
Evening/weekend	0.99 (0.97 – 1.01)	0.28
Night	0.90 (0.88 – 0.93)	<0.001
Season		
Spring	1.01 (0.98 – 1.03)	0.51
Summer	REF	
Fall	0.97 (0.95 – 1.00)	0.02
Winter	1.05 (1.03 – 1.07)	<0.001

CI = Confidence Interval; ISS = Injury Severity Score; MVC = Motor Vehicle Collision; GSW = Gunshot wound

© 2025 Tillmann BW et al. *JAMA Network Open*.

eAppendix E. Results – Sensitivity Analyses

Table 1. Relationship between updated FTT guidelines and triage rates stratified by distance from trauma center and alternative definitions of exposure period on the

	Baseline period	Implementation Period		Post-Implementation Period	
	Annual change in rates, RR (95% CI)	Instantaneous impact of announcement, RR (95% CI)	Change in annual rates relative to baseline, RR (95% CI)	Instantaneous impact of full rollout, RR (95% CI)	Change in annual rates relative to baseline, RR (95% CI)
Restricted to those within 50 miles of a trauma center whose nearest hospital was a non-trauma center					
Presentation to a trauma center	1.00 (1.00 – 1.01)	0.96 (0.89 – 1.04)	1.12 (0.99 – 1.27)	1.04 (0.92 – 1.18)	0.85 (0.75 – 0.97)
Undertriage	1.01 (1.00 – 1.02)	0.95 (0.89 – 1.02)	1.05 (0.93 – 1.18)	0.86 (0.76 – 0.96)	0.96 (0.85 – 1.08)
Population-level overtriage	1.03 (1.01 – 1.04)	0.87 (0.77 – 0.99)	1.26 (1.04 – 1.54)	0.91 (0.75 – 1.11)	0.73 (0.60 – 0.89)
Trauma center-level overtriage	1.01 (1.00 – 1.02)	0.91 (0.83 – 0.99)	1.14 (0.98 – 1.32)	0.85 (0.74 – 0.99)	0.86 (0.74 – 0.99)
Classifying all patients injured on or after July 1, 2014, as being treated in the post-implementation period					
Presentation to a trauma center	1.00 (0.99 – 1.01)	N/A	N/A	1.06 (1.03 – 1.10)	0.97 (0.96 – 0.98)
Undertriage	1.01 (1.00 – 1.02)	N/A	N/A	0.93 (0.91 – 0.96)	1.00 (0.99 – 1.01)
Population-level overtriage	1.01 (1.00 – 1.03)	N/A	N/A	1.04 (1.00 – 1.08)	0.96 (0.95 – 0.98)
Trauma center-level overtriage	1.00 (1.00 – 1.01)	N/A	N/A	0.97 (0.94 – 1.00)	0.99 (0.98 – 0.99)
Classifying all patients injured up until May 31, 2015, as being treated in the pre-implementation period					

© 2025 Tillmann BW et al. *JAMA Network Open*.

Presentation to a trauma center	1.00 (0.99 – 1.01)	N/A	N/A	1.10 (1.06 – 1.13)	0.96 (0.95 – 0.97)
Undertriage	1.01 (1.00 – 1.01)	N/A	N/A	0.91 (0.89 – 0.94)	1.02 (1.01 – 1.03)
Population-level overtriage	1.01 (1.00 – 1.02)	N/A	N/A	1.07 (1.02 – 1.11)	0.95 (0.94 – 0.96)
Trauma center-level overtriage	1.00 (1.00 – 1.01)	N/A	N/A	0.97 (0.94 – 0.996)	0.99 (0.98 – 1.00)

© 2025 Tillmann BW et al. *JAMA Network Open*.

Table 2. Case-mix adjusted annual trend in triage rates before and after FTT update stratified by distance from trauma center and alternative definitions of exposure period

	Baseline period	Post-Implementation Period
	EAPC (95% CI)	EAPC (95% CI)
Restricted to those within 50 miles of a trauma center whose nearest hospital was a non-trauma center		
Presentation to a trauma center	0.5% (-0.5 – 1.5%)	-4.0% (-5.0 – -3.1%)
Undertriage	0.9% (0.0 – 1.8%)	1.6% (0.6 – 2.5%)
Population-level overtriage	2.6% (1.1 – 4.1%)	-5.1% (-6.6 – -3.6%)
Trauma center-level overtriage	0.8% (-0.3 – 1.9%)	-1.7% (-2.9 – 0.5%)
Classifying all patients injured on or after July 1, 2014, as being treated in the post-implementation period		
Presentation to a trauma center	0.1% (-0.6 – 0.9%)	-2.6% (-3.2 – -2.0%)
Undertriage	1.0% (0.2 – 1.8%)	1.3% (0.7 – 1.9%)
Population-level overtriage	1.5% (0.4 – 2.5%)	-2.3% (-3.2 – -1.5%)
Trauma center-level overtriage	0.4% (-0.4 – 1.1%)	-0.6% (-1.3 – 0.1%)
Classifying all patients injured up until May 31, 2015, as being treated in the pre-implementation period		
Presentation to a trauma center	0.0% (-0.5 – 0.6%)	-4.2% (-4.9 – -3.4%)
Undertriage	0.7% (0.1 – 1.3%)	2.4% (1.7 – 3.2%)
Population-level overtriage	1.1% (0.3 – 1.9%)	-3.9% (-5.0 – -2.8%)
Trauma center-level overtriage	0.2% (-0.4 – 0.8%)	-0.4% (-1.2 – 0.5%)

EAPC = Estimated annual percent change

© 2025 Tillmann BW et al. *JAMA Network Open*.

Table 3. Relationship between update to FTT guidelines and undertriage stratified by definition of severe injury

	Baseline period	Implementation Period		Post-Implementation Period	
	Annual change in rates, RR (95% CI)	Instantaneous impact of announcement, RR (95% CI)	Change in annual rates relative to baseline, RR (95% CI)	Instantaneous impact of full rollout, RR (95% CI)	Change in annual rates relative to baseline, RR (95% CI)
Primary analysis	1.01 (1.00 – 1.02)	0.95 (0.89 – 1.01)	1.07 (0.97 – 1.18)	0.85 (0.77 – 0.94)	0.95 (0.86 – 1.05)
Expanded definition of severe injury	1.01 (1.00 – 1.01)	0.98 (0.95 – 1.01)	1.02 (0.97 – 1.07)	0.93 (0.89 – 0.98)	0.99 (0.94 – 1.04)
Components of undertriage definition					
ISS ≥ 16	1.01 (1.00 – 1.02)	0.94 (0.88 – 1.00)	1.08 (0.97 – 1.19)	0.84 (0.76 – 0.93)	0.94 (0.85 – 1.05)
Death within 24hrs	1.00 (0.96 – 1.05)	1.05 (0.75 – 1.47)	1.02 (0.58 – 1.81)	1.00 (0.57 – 1.74)	0.98 (0.56 – 1.73)
ACS identified injury	1.01 (1.00 – 1.01)	0.97 (0.93 – 1.00)	1.04 (0.98 – 1.10)	0.92 (0.87 – 0.97)	0.97 (0.92 – 1.03)
Mechanical ventilation in an ED	0.99 (0.97 – 1.01)	1.04 (0.90 – 1.19)	0.92 (0.72 – 1.18)	1.04 (0.82 – 1.32)	1.10 (0.86 – 1.41)
Blood within 24hrs	1.00 (1.00 – 1.01)	0.94 (0.89 – 1.00)	1.09 (0.99 – 1.20)	0.89 (0.81 – 0.97)	0.92 (0.84 – 1.02)

ACS = American College of Surgeons; ED = Emergency department

Table 4. Case-mix adjusted annual trend in undertriage rates before and after FTT update stratified by undertriage definition

	Baseline period		Post-Implementation Period	
	Crude undertriage rate	Case-mix Adjusted EAPC (95% CI)	Crude undertriage rate	Case-mix Adjusted EAPC (95% CI)
Primary analysis	63.6%	1.0% (0.2 – 1.8%)	63.3%	2.4% (1.7 – 3.2%)
Expanded definition of severe injury	72.8%	0.6% (0.2 – 0.9%)	73.7%	1.8% (1.4 – 2.1%)
Components of undertriage definition				
ISS ≥ 16	63.2%	1.1% (0.3 – 1.9%)	62.8%	2.7% (1.8 – 3.5%)
Death within 24hrs	63.1%	0.2% (-3.9 – 4.5%)	65.6%	1.0% (-3.4 – 5.6%)
ACS identified injury	59.8%	0.8% (0.3 – 1.3%)	63.4%	1.7% (1.3 – 2.2%)
Mechanical ventilation in an ED	56.8%	-0.8% (-2.6 – 1.0%)	52.7%	0.9% (-1.0 – 2.9%)
Blood within 24hrs	72.3%	0.4% (-0.2 – 1.1%)	71.1%	1.4% (0.7 – 2.2%)

EAPC = Estimated annual percent change; ACS = American College of Surgeons; ED = Emergency department

© 2025 Tillmann BW et al. *JAMA Network Open*.
