# Supplementary material for: Triage Accuracy and the 2015 Field Trauma Triage Criteria Update
Source: JAMA Netw Open. 2026 Jan 5;9(1):e2552092. doi: 10.1001/jamanetworkopen.2025.52092 (PMC12771241; doi:10.1001/jamanetworkopen.2025.52092)
Supplement: Supplement 2. — Data Sharing Statement [file jamanetwopen-e2552092-s002.pdf]

Data Sharing Statement

Tillmann. Triage Accuracy and the 2015 Field Trauma Triage Criteria Update. *JAMA Netw Open*. Published January 05, 2026. doi:10.1001/jamanetworkopen.2025.52092

Data
Data available: No

Additional Information
Explanation for why data not available: The dataset from this study is held securely in coded form at ICES. While legal data sharing agreements between ICES and data providers (e.g., healthcare organizations and government) prohibit ICES from making the dataset publicly available, access may be granted to those who meet pre-specified criteria for confidential access, available at www.ices.on.ca/DAS (email: das@ ices.on.ca).
